# Physicochemical and Functional Properties of Modified KJ CMU-107 Rice Starches as Pharmaceutical Excipients

**DOI:** 10.3390/polym14071298

**Published:** 2022-03-23

**Authors:** Ornanong S. Kittipongpatana, Nisit Kittipongpatana

**Affiliations:** 1 Department of Pharmaceutical Sciences, Faculty of Pharmacy, Chiang Mai University, Chiang Mai 50200, Thailand; ornanong.kit@cmu.ac.th; 2 Lanna Rice Research Center, Chiang Mai University, Chiang Mai 50100, Thailand

**Keywords:** rice starch, chemical modification, pre-gelatinization, physicochemical property, pharmaceutical excipient

## Abstract

Starch extracted from KJ CMU-107 rice, with amylose content of 13.4%, was modified to yield pre-gelatinized starch (PGS), carboxymethyl starch (CMS), crosslinked carboxymethyl starch (CLCMS), crosslinked starch (CLS), and hydroxypropyl starch (HPS). Their physicochemical properties were assessed in comparison with the native starch (NS), and their functional properties were then evaluated for potential use as pharmaceutical excipients. Scanning electron microscopic (SEM) images and X-ray diffraction (XRD) patterns showed that granules of all but one of the modified starches retained the native character and crystalline arrangement. The exception, PGS, exhibited extensive granular rupture, which correlated with the loss of crystallinity suggested by the amorphous halo in XRD. Energy-dispersive X-ray (EDX) data confirmed the modification by the presence of related elements. Carboxymethylation increased solubility in unheated water, while crosslinking improved swelling. All modified starches displayed improved oil absorption capacity by 17–64%, while CMS and CLCMS also exhibited significant moisture sorption at above 75% RH PGS and HPS exhibited lower gelatinization temperature (Tg) and enthalpic change (ΔH), while CLS showed higher Tg and ΔH. CMS, CLCMS, and CLS showed adequate powder flow and compactibility, qualifying as potential tablet excipients. The 5% *w*/*v* solutions of CMS, CLMS, and HPS also formed intact films with suitable tensile strength. Overall, modified starches derived from KJ CMU-107 could potentially be developed into new pharmaceutical excipients.

## 1. Introduction

Starches have long been utilized as pharmaceutical excipients, especially in the solid dosage form. Due to its availability, inertness, and low cost, native starch powder has been used as a diluent as well as a disintegrant in tablets. Starch paste has also been applied as a binder to facilitate the manufacturing of pharmaceutical tablets [1,2]. Commercial starches are derived from various plants and plant parts, including roots (cassava, arrowroot), tubers (potato, yam), and cereal grains (wheat, corn, rice). The impacts of starch sources on physicochemical, mechanical, and pharmaceutical properties and applications have been widely studied and well documented [3,4]. The differences in characteristics such as granule shape and size, amylose content, and crystallinity type, among others, contribute to variations in the functional properties of each starch. Further modification of native starch by physical, chemical, or enzymatical techniques alters the structure and physicochemical properties and, in turn, yields modified starches with broadened functional properties and applications [5,6]. Modified starches are now commonly found not only as ingredients in foods [7,8], but also as compositions or additives in cosmetics [9,10] and excipients in pharmaceuticals [1,3].

Rice (*Oryza sativa* L.) is one of the major starch-producing food crops. With the 2020 worldwide market value of rice starch reported at over 201.6 million USD, and the demand trending upward for applications in food, pharmaceutical, cosmetics, and other industries [11], it is suggested that excellent opportunities await the development of new products from rice starch. As a number of new rice varieties continuously become available via breeding research and development, the preparation and investigation of the subsequent modified rice starches also increase [12,13]. A major advantage of rice starch over other starches includes its broad range of amylose content (0–35%) from different rice varieties, its unique polygonal shape which allows interlocking between granules during compaction, as well as its inherited characteristics from the breeding process, such as color and odor. Kum Jao Morchor 107 (KJ CMU-107), a purple pericarp, lowland rice variety, is the product of a cross between the purple glutinous rice variety Kum Doi Saket and the white non-glutinous rice variety Jasmine-105 [14]. Due to its aroma property and presence of anthocyanins with antioxidant activity [15], this Thai rice variety is considered a special quality rice for consumption, with potential value as a nutraceutical, pharmaceutical excipient, and cosmetic ingredient.

In this study, we prepared a physically modified starch and four chemically modified starches from KJ CMU-107 rice starch. The physicochemical and functional properties of the modified starches were evaluated, in comparison with those of native starch, to assess their potential as pharmaceutical excipients.

## 2. Materials and Methods

### 2.1. Materials

KJ CMU-107 rice grains were provided by Lanna Rice Research Center (Chiang Mai, Thailand). Monochloroacetic acid (MCA, CAS No. 79-11-8, product code 8004121000) was purchased from Merck (Hohenbrunn, Germany). Sodium trimetaphosphate (STMP, CAS No. 7785-84-4, product code 1001229448) and propylene oxide (PO, CAS No. 75-56-9, product code 101920564) were supplied by Aldrich (Wyoming, IL, USA). All other chemicals employed in the preparation and analysis of modified starches were of AR grade or equivalent.

### 2.2. Extraction of Starch

The preparation of the flour from the rice grains was carried out by the conventional wet-milling method. For starch extraction, 700 mL of sodium hydroxide (NaOH, 0.05 M) was mixed with a 250 g portion of rice flour and mechanically stirred for 5 h at room temperature. The suspension was subsequently filtered through a 75 μm wire mesh, and the slurry was allowed to settle for 12 h. The clear supernatant was discarded, and the sediment was washed three times with distilled water. The pH of the mixture was adjusted to 7.0 with 1 M hydrochloric acid (HCl), allowed to settle, and the supernatant was removed. The extracted starch was washed several more times with water (600 mL each) and was finally collected via vacuum filtration. Following an air-dry and a subsequent oven-dry at 40 °C for 12 h, the native starch powder was ground to pass through sieve no. 60 (60 mesh, 0.250 mm) before use.

### 2.3. Preparation of Modified Rice Starches

**Figure 1:**
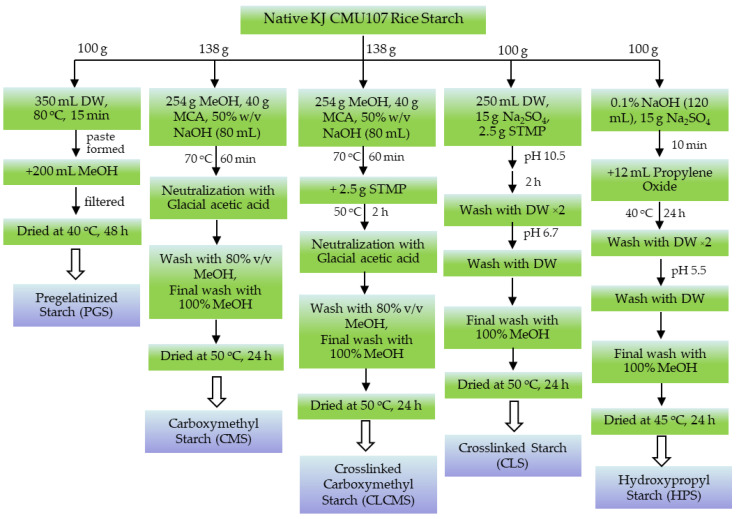
Synthesis Schemes for the Preparation of Modified Starches.

Five modified rice starches, namely, pre-gelatinized starch (PGS), carboxymethyl starch (CMS), crosslinked carboxymethyl starch (CLCMS), crosslinked starch (CLS), and hydroxypropyl starch (HPS), were prepared according to the scheme shown in Figure 1.

For CMS and CLCMS, the degree of substitution (DS) of carboxymethyl groups was determined as described previously [16]. The degree of phosphate crosslinking (DCx) for CLCMS and CLS was determined as described previously [17]. The hydroxypropyl content of HPS was analyzed by spectrophotometry and reported in terms of molar substitution [18].

### 2.4. Physicochemical Property Evaluation

#### 2.4.1. Amylose Content

The amylose content (AC) of native starch was determined according to the method described by Gibson et al. [19], using an Amylose/Amylopectin assay kit (K-AMYL, Megazyme, Ireland). The procedure was based on the elimination of amylopectin via the specific formation of amylopectin complexes with the lectin concanavalin A. The AC was then measured spectrophotometrically after hydrolysis to glucose.

#### 2.4.2. Scanning Electron Microscopic (SEM) with Energy-Dispersive X-ray Analysis

SEM studies to visualize the granule surface, shape, and size were conducted using a JEOL instrument model JSM-5410LV (JEOL, Tokyo, Japan) equipped with a tungsten filament K-type. The acceleration voltage was 15 kV under the low vacuum mode (0.7–0.8 torr). The sample was mounted on a copper stub covered with carbon tape and sputter-coated with gold. The magnification was set at 2000×. The analysis of chemical composition was performed using an energy-dispersive X-ray (EDX) spectrometer (ISIS 300, Oxford Instrument, High Wycombe, UK).

#### 2.4.3. X-ray Diffraction

X-ray diffraction (XRD) analysis of starch and modified starch samples was carried out using a Siemens D-500 X-ray diffractometer. Diffractograms were recorded in the Bragg Angle (2θ) range of 5° to 40°. The scan rate was 2.5°/min, and the step size was 0.02°.

#### 2.4.4. Water Solubility and Swelling Power at 70 °C

Starch samples (0.1 g) were accurately weighed into a pre-weighed centrifuge tube. Then, 10 mL water was added, the mixture was vortexed for 1 min, and then heated at a controlled temperature of 70 °C with regular stirring. After 10 min, the tube was cooled and centrifuged at 3000× *g* for 15 min. The clear supernatant was transferred to a preweighed crucible and dried at 120 °C to a constant weight, which was used to calculate the solubility percentage. The weight of the water-absorbing sediment was used to determine the swelling power. The process was repeated 5 times for each sample.

#### 2.4.5. Free-Swelling Capacity at 37 °C

The teabag method described by Heß et al. [20] was used to determine the free swelling capacity (FSC), with minor modifications. First, a 1.0 g sample was accurately weighed into a dry, pre-weighed teabag. The bag was sealed and placed in a temperature-controlled water bath, set at 37 °C. After 15 min, the bag was removed from the water and suspended above the bath to drain out the excess water from the teabag. The weight of the teabag and the swelling content was then determined, and the FSC value (*q*) was calculated as follows:

$$q_{\mathrm{FSC}}=\frac{m_{t}-m_{tb}-m_{w}}{m_{s}}$$

where *m_t_* is the total weight of the teabag and the swelling content; *m_tb_* is the weight of the empty, dry teabag; *m_w_* is the weight of the water absorbed by the empty teabag; and *m_s_* is the weight of the dry sample.

#### 2.4.6. Moisture Content

The moisture content of the starch sample was determined using an Ohaus MB25 moisture content balance (Ohaus Corp., Parsippany, NJ, USA) equipped with a halogen radiator. Approximately 2 g of the sample was spread on the pan and the accurate weight was recorded. The sample was heated at 105 °C until a constant weight was obtained. The moisture content was then calculated as the difference between the two weights divided by the original weight of the sample.

#### 2.4.7. Moisture Sorption

The moisture sorption of starch samples was assessed for 7 days at various relative humidity (RH) levels. One gram of each sample, previously dried for 4 h at 80 °C, was accurately weighed into a pre-weighed, 2.5 cm diameter cup. The cups were then stored in desiccator housing beakers of saturated salt solutions, which provided 51, 62, 75, 83, and 92% RH at 30 °C for 7 days. The weight differences on day 0 and day 7 were determined and used in the calculation of moisture sorption of samples in terms of the percentage of weight gained.

#### 2.4.8. Oil Absorption Capacity (OAC)

OAC analysis employed a method described by Bhosale and Singhal [21], with some modifications. The starch sample (0.1 g) was accurately weighed in a microcentrifuge tube. Mineral oil (1 g) was added, and the tube was weighed again before being mixed vigorously with a vortex mixer for 1 min. After settling for 30 min at room temperature, the tube was centrifuged at 4000× *g* for 20 min. The unabsorbed oil at the top of the tube was discarded, and the weight of the oil-absorbing sample was determined. The OAC value was calculated and reported as gram of oil absorbed per gram of starch sample.

#### 2.4.9. Thermal Properties

Thermal properties were evaluated using a DSC-7 differential scanning calorimeter (Perkin Elmer Inc., Branford, CT, USA). The analysis was carried out at a temperature between 30 °C and 120 °C, at a rate of 10 °C/min. Each sample pan contained 3 mg of starch and 15 μL of water. An empty pan was used as a reference. The onset temperature (T_o_), peak temperature (T_p_), and conclusion temperature (T_c_) were recorded. The temperature range (T_c_ − T_o_, ΔT) of each sample was calculated. The enthalpy (ΔH) of gelatinization transition was expressed as J/g of dry starch.

### 2.5. Pharmaceutical Functionality Evaluation

#### 2.5.1. Density and Powder Flow

The bulk and tapped densities of starch samples were determined according to the standard USP method [22]. Carr’s index (CI) and the Hausner ratio (HR) were then calculated using the following equations:

Carr’s index = (Tapped density − Bulk density)/Tapped density × 100

Hausner ratio = Tapped density/Bulk density

The powder flow was assessed using the fixed funnel method and the angle of repose (AR) calculation. In brief, the sample (100 g) was poured through a 15 cm diameter glass funnel held at a height of 15 cm onto a piece of paper on a level bench top. As the sample formed a conical pile, its height (h) and radius (r) were determined. The inverse tangent of the h/r ratio yielded the angle of repose.

#### 2.5.2. Powder Compactibility

The starch sample (250 mg) was filled into a 4.6 mm flat-face punch and die set, which was then compressed using a hydraulic press (Carver Inc., Wabash, IN, USA) at compression forces of 0.5, 1.0, 1.5, and 2.0 tons. The hardness of the resulting tablet was measured in triplicate using a hardness tester (Erweka, Langen, Germany). A pressure-hardness profile (PHP) was plotted between the compression force applied and the hardness of the tablets.

#### 2.5.3. Film Forming Ability and Tensile Strength Test

Starch samples (5 g) were dispersed in 100 mL deionized water while stirring at 150 rpm using a magnetic stirrer (Heidolph Instruments, Schwabach, Germany). For NS, PGS, CLS, and HPS, heat was applied to facilitate gelatinization, while CMS and CLCMS were dissolved in unheated water. Each solution was degassed, and 30 mL was poured on a Teflon plate and then dried in a hot-air oven at 50 °C for 16 h. The film-forming ability of each starch sample was inspected visually. Intact films were stored in a humidity chamber (25 °C, RH = 67%) for mechanical tests. Tensile strength (MPa) and elongation at break percentage (%) of the intact films were determined, in triplicates, using a TA-XT Plus Texture Analyzer (Stable Micro Systems, Surrey, UK) according to the ASTM standard method (D882-02, 2002) with slight modifications. Intact films were cut into rectangular strips (2 × 6 cm) and stored at 25 °C and 58% RH for 48 h before testing. The initial grip separation and crosshead speed were set to 50 mm and 0.5 mm/s, respectively.

### 2.6. Statistical Analysis

All tests were carried out in triplicate, unless indicated otherwise, and the data are presented as average values. Statistical analysis was conducted using a one-way analysis of variance (ANOVA) in SPSS (version 19.0). Tukey’s honestly significant difference (HSD) multiple range test was used to determine significance at a 95% confidence level (*p* < 0.05).

## 3. Results and Discussion

### 3.1. Amylose Content and General Appearances

The amylose content (AC) of native KJ CMU-107 rice starch was determined to be 13.4%, which was classified into the group of low-amylose rice starch (10–19% AC). This value, which was determined by the lectin concanavalin A procedure, was slightly lower than the 14% AC previously reported for this rice strain [15]. Pre-gelatinization of starch, carried out in starch suspension using heat and mechanical stirring, resulted in the formation of white, viscous paste, indicating the change in the structural integrity of starch granules. Methanol was added to cease the process, preventing the starch from becoming excessively gelatinized, which would otherwise lead to the degradation of the starch chain and a decrease in water absorption ability [23]. Carboxymethylation of native starch yielded a product that visibly formed gel upon contact with water. The degree of carboxymethyl substitution (DS) was determined to be 0.24 ± 0.03. The dual-modified, carboxymethyl-crosslinked starch exhibited the same property, but the gel was less viscous. The DS was determined to be 0.20 ± 0.02, while the degree of crosslinking (DCx) was calculated as 0.022 ± 0.001. The crosslinked starch and the hydroxypropyl starch showed no remarkable difference compared to the native starch. CLS yielded a DCx of 0.026 ± 0.001, while the molar hydroxypropyl substitution (MS) of HPS was 0.019 ± 0.002. The higher DS observed in CMS compared to CLCMS and the higher DCx obtained from CLS versus CLCMS were consistent with the previous results which suggested that the two reactions targeted similar sites on the starch chains. Both reactions were reported to occur at the -OH groups at O-2 and O-6 much more than at O-3, with the carboxymethylation taking place at O-2 > O-6 and phosphate crosslinking slightly preferring O-6 > O-2 [24,25]. The MS value of the prepared HPS was consistent with the commercial, low-molar substituted HPS product [26].

### 3.2. SEM and EDX

**Figure 2:**
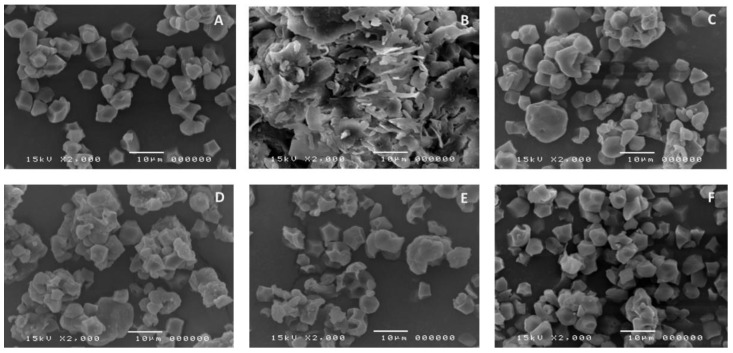
SEM Images of (**A**) Native, (**B**) Pre-gelatinized, (**C**) Carboxymethyl, (**D**) Crosslinked Carboxymethyl, (**E**) Crosslinked, and (**F**) Hydroxypropyl Starches Derived from KJ CMU-107 Rice Starch. Images Taken at 2000× Magnification.

Granules of native KJ CMU-107 rice starch (NS) were mainly polygonal with diameter sizes ranging from 2 to 6 μm. Some granules appeared agglomerated, forming large, irregular shape mass with a slightly rough, indented surface. This was likely due to damage to the starch during the extraction process (Figure 2A). SEM of PGS (Figure 2B) showed an extensive loss of the granular structure, as indicated by irregular surface, plate-like structures along with some small, fragmented debris. This can be attributed to the heat employed in the pre-gelatinization process, which caused the granules to swell and rupture [27]. Granules of CMS (Figure 2C) and, in particular, CLCMS (Figure 2D) formed agglomerates, probably due to their hygroscopicity. SEMs of CLS (Figure 2E) and HPS (Figure 2F) showed that most granules appeared intact, evenly distributed in both shape and size, and were not remarkably different from those of native starch.

**Figure 3:**
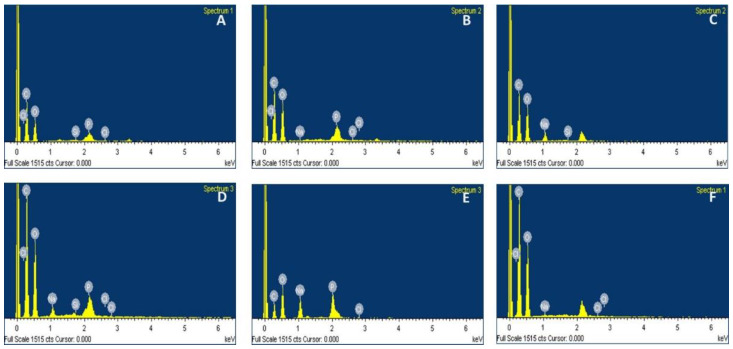
EDX Spectra of (**A**) NS, (**B**) PGS, (**C**) CMS, (**D**) CLCMS, (**E**) CLS, and (**F**) HPS Modified from KJ CMU-107 Rice Starch.

The energy-dispersive X-ray (EDX) data and spectra of the native and modified rice starches are presented in Table 1 and Figure 3A–F, respectively. EDX analysis provides the identity of the chemical compositions as well as their percentage in the sample, except hydrogen, which was non-detectable with this method. The gold elemental peak (Au) was omitted from each spectrum as it was introduced to the sample during the sputter coating. The spectrum of native starch (Figure 3A) showed the presence of carbon (C) and oxygen (O) as main elemental compositions at the weight ratio of 1.2 to 1, while a small amount of phosphorous was also detected. Rice starch was previously reported to contain high organic phosphorous content from the residual ash of phospholipids, compared to other starch sources [28,29]. A similar profile and C to O ratio was observed for PGS, with slightly lower phosphorous (P) content (Figure 3B). Chemically modified starch exhibited significantly different profiles. CMS yielded the C to O ratio of 1.1 to 1, with an increase in sodium (Na) content (Figure 3C) as part of the structure (as sodium carboxymethyl starch or sodium starch glycolate). Both CLCMS (Figure 3D) and CLS (Figure 3E) showed similar C to O ratios at 0.9 to 1, with increased P contents of 1.12% and 1.8%, respectively, as a result of the formation of phosphodiester linkages of the crosslinking structure. Lower P content observed in CLCMS suggests that the crosslink reaction was interfered by the presence of carboxymethyl groups from the carboxymethylation, which was consistent with the lower DCx calculated for CLCMS compared to CLS. The higher Na content (3.09%) in CLCMS compared to CMS was due to the salt formation in the negatively charged oxygen atoms in the carboxymethyl groups and the phosphate groups on the starch polymer. HPS (Figure 3F) possessed the highest C to O ratio at >1.3 to 1 as the hydroxypropyl group was incorporated into the structure. The low chloride (Cl) content detected in all samples indicated that NaCl, a by-product of the washing steps, was mostly eliminated.

### 3.3. X-ray Diffraction

**Figure 4:**
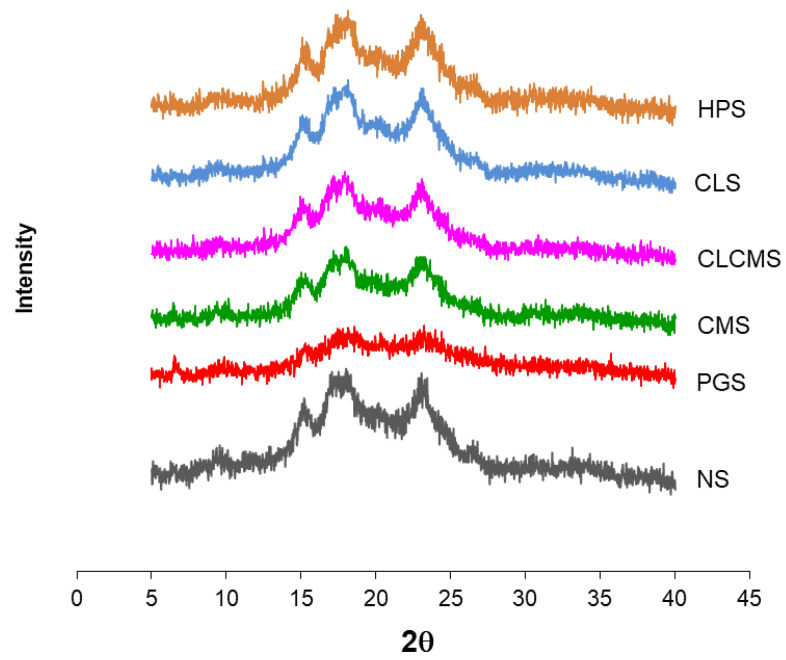
XRD Profiles of Native and Modified KJ CMU-107 Starches.

Native KJ CMU-107 rice starch exhibited a typical A-type XRD pattern, with major diffraction peaks at Bragg angles (2θ) of 15.1, 17.9, and 23.0 (Figure 4). PGS showed significant loss of crystallinity as all three major peaks became almost undetectable. The chemically modified starches retained most of their crystalline structure, with CMS and CLCMS being affected more than the other two modified starches.

### 3.4. Swelling Power and Solubility

**Figure 5:**
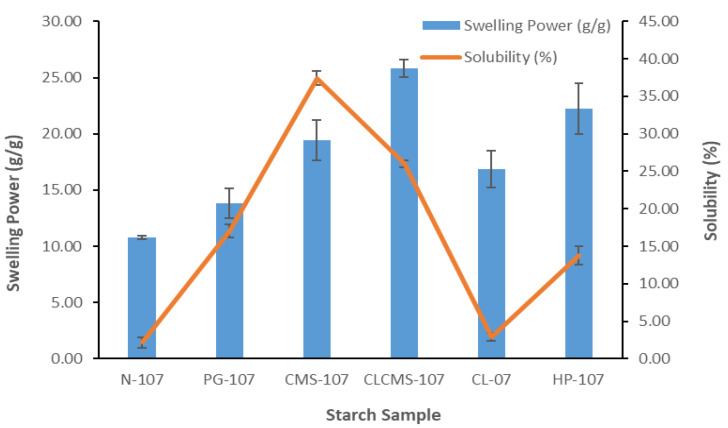
Swelling Power (*g*/*g*) and Solubility (%) of Native and Modified KJ CMU-107 Rice Starches Determined at 70 °C.

The swelling power and the solubility of each starch, assessed at 70 °C, are displayed in Figure 5. The swelling power of starch samples was in the descending order of CLCMS > HP > CMS > CLS > PGS > NS, while the solubility was in the descending order of CMS > CLCMS > PGS > HPS > CLS > NS. As expected, native KJ CMU-107 starch exhibited low swelling power and solubility. The hydrogen bonds between starch chains, together with the amylose–amylopectin and amylopectin–amylopectin interactions, prevented water penetration into the granules [23]. For comparison purposes, NS possessed better swelling power but exhibited less water solubility compared to the commercial cassava starch (CAS) (Table 2). Both parameters were markedly improved as the starch was subjected to pre-gelatinization. This was in part because the ruptured PGS granules presented greater surface area for water absorption. Granule swelling also led to the leaching of amylose into the surrounding water, resulting in increased solubility [30,31]. Phosphate crosslinking of NS improved the swelling power by 1.5-fold. This result is in contrast with previous studies in which crosslinking impeded the swelling of starch by forming intermolecular bridges between starch chains, which immobilized the bond flexibility and limited molecular mobility [17,32]. In this study, it is proposed that the crosslinking reaction disrupted the hydrogen bonds between starch molecules, thus allowing the infiltration of water into starch granules. However, the rigidity of crosslinked bonds retarded the free swelling, and the swelling at low temperature occurred only slightly more than that of the native starch, as evidenced by the FSC values. At high-temperature heating, the crosslinked bonds maintained the integrity of the granules by preventing the burst while the granules continued to absorb water, resulting in an increase in swelling power. Carboxymethylation or hydroxypropylation yielded modified starches with higher swelling power, as well as increased solubility. The carboxymethyl group is hydrophilic and the substitution of hydroxyl groups with carboxymethyl groups rendered the starch molecules soluble/swelling in cold water and exerts potential as a gelling agent, a tablet binder, or a disintegrant [16,17,20,24,33]. The ability of hydroxypropylation to facilitate the swelling of starch granules has previously been reported [34,35]. The hydroxypropyl substitution induced the repulsion between starch chains, thus attenuating the molecular hydrogen bonding and allowing the perforation of water into the granules, which enhanced solubility [35] and could serve as a tablet disintegrant [26]. Dual-modified CLCMS displayed higher swelling power and lower solubility compared to the non-crosslinked CMS. It is postulated that the formation of crosslinked bonds maintained the structural integrity of the swelling CMS granules by creating a strong gel network that continually absorbed and entrapped water inside, which led to the higher swelling power. Consequently, fewer starch molecules were found in the water outside of the network, which resulted in the lower solubility of the CLCMS.

### 3.5. Free Swelling Capacity

Free swelling capacity (FSC) of native and modified starches, determined at 37 °C after 15 min, yielded results in the order of CLCMS > CMS > CLS > PGS > NS ~ HP (Table 2). This parameter served as a suitable indicator of potential tablet disintegrating excipients, as swelling is one of the most widely accepted mechanisms for tablet disintegration [36]. CLCMS and CMS represent two favorable candidates due to the high FSC values at 37 °C. FSC results also presented suitable correlation with the swelling power determined at 80 °C, with the exception of HPS, which exhibited significant swelling power at high-temperature heating, but the FSC value at 37 °C was only comparable to that of NS. This result suggests that HPS would not be an effective tablet disintegrant and would likely require additional modification, e.g., pre-gelatinization to improve its potential as an excipient [26].

### 3.6. Oil Absorption Capacity

OAC of starch represents a suitable measurement of its potential as an emulsifying agent [37]. The OAC value was improved as the native starch (OAC = 1.68 *g*/*g*) was physically modified into pre-gelatinized starch (OAC = 1.97 *g*/*g*). A higher particle surface area due to the physical disruption of PGS granules was likely the main contributor to this result. Modification of starch by carboxymethylation or crosslinking reactions significantly increased the OAC, while a combination of both reactions further increased the value to 2.76 *g*/*g*. The OAC value was also increased in the case of hydroxypropylated starch (Table 2). The higher absorption observed in all chemically modified starches could partially be attributed to the interaction between the substituted groups and the oil. The increases in OAC were also reported from carboxymethyl starch of papaya [38], hydroxypropyl starch of water yam [39], and crosslinked carboxymethyl mungbean starch [40].

### 3.7. Moisture Content and Moisture Sorption

**Figure 6:**
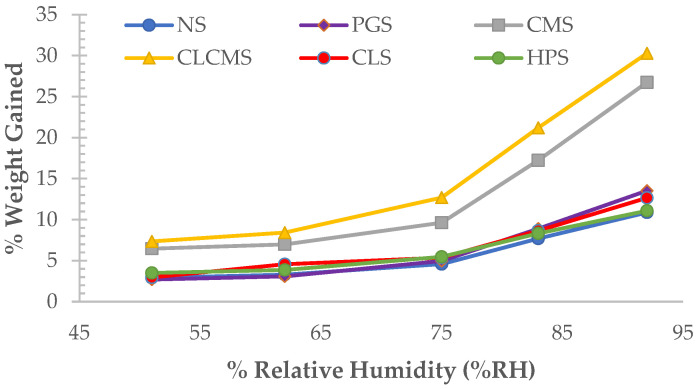
Moisture Sorption Profiles of Native (NS) and Five Modified KJ CMU-107 Rice Starches, Including Pre-gelatinized Starch (PGS), Carboxymethyl Starch (CMS), Crosslinked Carboxymethyl Starch (CLCMS), Crosslinked Starch (CLS), and Hydroxypropyl Starch (HPS).

NS and all but one of the modified starches exhibited less than 10% moisture sorption at relative humidity (RH) up to 75% (Figure 6). The exception was CLCMS, which gained 12.7% weight at this RH. At above 75% RH, the moisture sorption of NS, PGS, CLS, and HPS increased slightly but remained below 15%, while that of CMS and CLCMS rose significantly and were more than 25% at 92% RH. Carboxymethylation has been shown to increase the hygroscopicity of starch. The results suggest that carboxymethylation affected moisture sorption at high % RH. This is likely the result of increased hydrophilicity of the modified starch molecules in the (Na) salt form with carboxymethyl group substitution, which enhanced water vapor sorption. The addition of crosslinked bonds into the structure further enhanced the moisture uptake of starch granules, as evidenced by a significantly higher moisture sorption of CLCMS compared to CMS, as well as higher values observed in CLS compared to NS. It is possible that by forming crosslinked bonds between starch chains, an amylopectin-like structure was created. Amylopectin was reported to be more hygroscopic than amylose [23].

### 3.8. Thermal Properties

The onset temperature (T_o_), peak temperature (T_p_), conclusion temperature (T_c_), and the order–disorder transition enthalpy (ΔH) of KJ CMU-107 rice starch and modified starches are presented in Table 3. Gelatinization temperature (T_g_) of KJ CMU-107 native starch occurred at 72.1 ± 1.1 °C, with the enthalpy change (ΔH) was calculated at 8.5 ± 0.5 J/g. Both values were on the lower side compared to those reported for rice starch in other studies [41,42]. This was likely the influence of the amylose content (AC) of starch, as the amylose double helices required a high temperature and energy intake to forgo the ordered structure [43]. Starch with lower AC tended to have lower gelatinization temperatures [29]. PGS was previously exposed to heat and the ordered granular structures had been disrupted, thus requiring a lower temperature to gelatinize. The decrease in ΔH was consistent with the loss of molecular integrity within the pre-gelatinized starch granules [17]. CMS and CLCMS were soluble and/or swelling in cold water, and both showed no transition peak in the studied range. Similar results were previously reported for these modified starches prepared from other starch sources [33,36,40]. In CLS, the formation of crosslinking covalent bonds between starch chains strengthened the granules [29] and enhanced hydrogen bond interactions, which further decreased the chain mobility. These resulted in a higher gelatinization temperature and enthalpic change, as more energy was needed for granules to swell and rupture [44]. HPS gelatinized at a lower temperature than NS and PGS. The substitution of the hydroxyl groups with the bulkier hydroxypropyl groups abated the hydrogen bonds, causing the disruption of helical structures. This loss of molecular order was reflected by the decrease in ΔH. For comparison purposes, cassava starch (CAS) exhibited higher gelatinization temperatures and ΔH compared to native KJ CMU-107 starch.

### 3.9. Pharmaceutical Functionality

#### 3.9.1. Density and Powder Flow

All modified starches exhibited lower tapped and bulk densities compared to those of native starch (Table 4). Carr’s index and the Hausner ratio suggest that PGS possessed the worst powder flowability, which was confirmed by the angle of repose (AR) result. The flowability of pre-gelatinized starches was generally poor because of the disruption of granular structure into smaller particle sizes, with a higher specific surface area [45]. The better powder flow of CMS and CLCMS likely stemmed from the agglomeration of granules observed in SEM images. CLS and HPS yielded similar CI and HR values to native starch. The AR result, however, suggested that CLS flowed better than both NS and HPS. For comparison purposes, CAS possessed lower densities and inferior flowability compared to native KJ CMU-107 starch.

#### 3.9.2. Powder Compactibility

**Figure 7:**
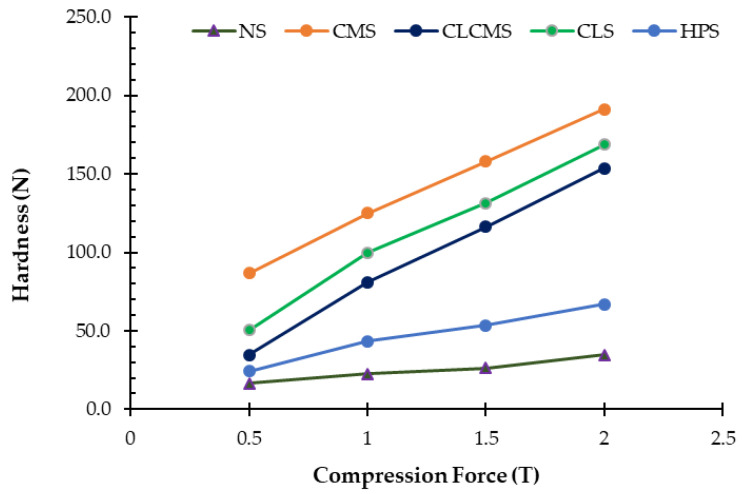
Pressure-hardness Relationship of Native and Modified Starches Prepared from KJ CMU-107 Rice Starch.

The pressure–hardness profile (PHP, Figure 7) represents the ability of starch powders to be compressed, with relationship to the force (T), into a tablet of measurable strength (N) [46]. PGS did not form tablets of significant strength and thus was not included in the comparison. Figure 6 shows that the native starch had the lowest compactibility among all starches. The initial hardness was low (16 N at 0.5 T) and did not increase proportionally with the increased compression force. HPS exhibited a slightly better profile, but the maximum tablet hardness remained under 70 N at 2 T compression force. In contrast, CLS, CLCMS, and CMS yielded decent PHP profiles, with the maximum tablet hardness above 150 N at 2 T compression force. Native rice starch, with the polygonal granule shape, was reported to have better consolidation upon compression compared to other starches with rounder granules (i.e., corn, tapioca, mungbean) due to particle inter-locking [47]. As the granules were ruptured and the particles became smaller and varied in shape, such particle interlocking no longer dominated, and the compaction of PGS into tablets was not possible.

### 3.10. Film Forming Property

**Figure 8:**
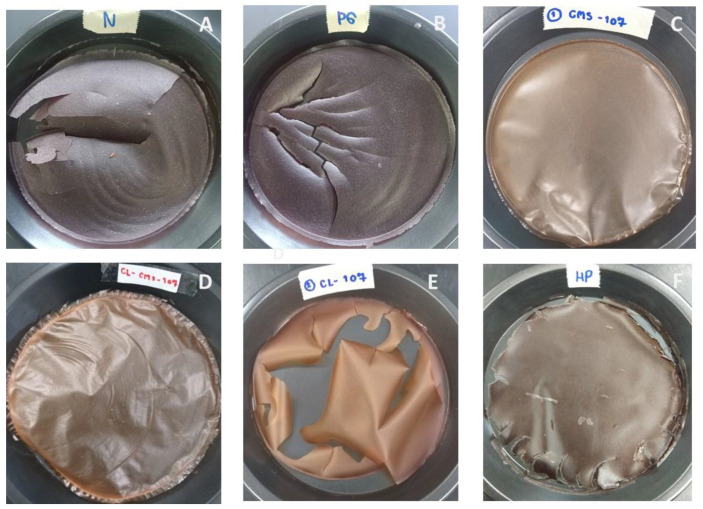
Films Prepared from (**A**) NS, (**B**) PGS, (**C**) CMS, (**D**) CLCMS, (**E**) CLS, and (**F**) HPS. Casting Solution Contained 5% *w*/*v* Starch; Drying Was at 50 °C for 16 h.

CMS, CLCMS, and HPS formed intact films upon casting the 5% *w*/*v* solution on Teflon plates. CMS film, in particular, exhibited a smooth surface with adequate flexibility. In contrast, NS, PGS, and CLS films were brittle and easily broken (Figure 8).

The TS and Eb were determined on the three intact films. For CMS, CLCMS, and HPS, TS values were 20.1 ± 1.4, 24.3 ± 1.9, and 16.1 ± 2.1 MPa, and Eb values were 3.76%, 2.87%, and 1.22%, respectively. All three intact films shared a common feature in that the hydroxyl groups were substituted with the larger moieties, which were able to disrupt the inter- and intra-molecular hydrogen bonding in the starch polymer [48]. This increased the flexibility of the network that allowed the formation of film.

## 4. Conclusions

Physical and chemical modifications constitute effective means to optimize the physicochemical and functional properties of starch. From altering fundamental properties such as solubility, swellability, moisture sorption, and water and oil absorption to improving mechanical properties such as flowability, compactibility, film-forming ability, and film strength, modified rice starches offer opportunities for broad applications in the food, pharmaceutical, and cosmetic industries.

## Figures and Tables

**Table 1:** Energy-dispersive X-ray (EDX) Analysis of Native and Modified KJ CMU-107 Starches.

Sample	Element Weight (%)				
	C	O	Na	P	Cl
NS	54.19 ± 3.30	45.17 ± 3.53	0.02 ± 0.02	0.58 ± 0.71	0.02 ± 0.04
PGS	53.83 ± 1.04	45.80 ± 1.08	0.00 ± 0.01	0.31 ± 0.06	0.04 ± 0.03
CMS	51.54 ± 1.92	46.55 ± 1.68	1.81 ± 0.27	0.05 ± 0.08	0.01 ± 0.02
CLCMS	46.44 ± 3.29	49.29 ± 1.92	3.09 ± 1.19	1.12 ± 0.50	0.05 ± 0.06
CLS	46.10 ± 6.68	48.89 ± 2.92	3.17 ± 2.73	2.63 ± 0.95	0.02 ± 0.02
HPS	57.84 ± 1.45	42.03 ± 1.50	0.09 ± 0.00	0.02 ± 0.00	0.02 ± 0.02

**Table 2:** Powder Properties of Native and Modified KJ CMU-107 Starches.

Sample	MC (%)	SP @80 °C (*g*/*g*)	Solu (%)	FSC @37 °C (*g*/*g*)	OAC (*g*/*g*)
NS	9.48 ± 0.56 ^cd^	10.78 ± 0.15 ^f^	2.15 ± 0.74 ^e^	4.53 ± 0.03 ^e^	1.68 ± 0.11 ^e^
PGS	10.71 ± 1.03 ^ab^	13.79 ± 1.32 ^e^	17.06 ± 0.88 ^c^	5.95 ± 0.49 ^d^	1.97 ± 0.10 ^d^
CMS	10.95 ± 0.77 ^a^	19.44 ± 1.81 ^c^	37.41 ± 0.92 ^a^	14.51 ± 1.48 ^b^	2.18 ± 0.13 ^c^
CLCMS	11.70 ± 1.12 ^a^	25.85 ± 0.78 ^a^	26.00 ± 0.51 ^b^	17.79 ± 0.18 ^a^	2.76 ± 0.14 ^a^
CLS	9.93 ± 1.25 ^bc^	16.84 ± 1.65 ^d^	2.90 ± 0.50 ^e^	7.12 ± 0.68 ^c^	2.46 ± 0.20 ^ab^
HPS	9.01 ± 0.94 ^d^	22.24 ± 2.23 ^b^	13.78 ± 1.27 ^d^	4.34 ± 0.19 ^e^	2.30 ± 0.10 ^bc^
CAS	10.82 ± 0.84	8.81 ± 0.69	5.70 ± 0.88	4.17 ± 0.36	1.84 ± 0.28

Values under the same heading with identical superscript are not significantly different (*p* < 0.05).

**Table 3:** Thermal Properties of Native and Modified KJ CMU-107 Rice Starches.

Sample	Temperature (°C)			ΔT	ΔH (J/g)
	T_onset_	T_peak_	T_end_		
NS	64.3 ± 1.1 ^b^	72.1 ± 1.1 ^b^	78.6 ± 0.9 ^b^	14.6 ± 1.7 ^a^	8.5 ± 0.5 ^b^
PGS	64.5 ± 0.7 ^b^	70.9 ± 0.3 ^c^	76.5 ± 1.1 ^c^	12.3 ± 0.8 ^b^	6.0 ± 0.6 ^d^
CMS	N/A				
CLCMS	N/A				
CLS	67.1 ± 0.7 ^a^	74.9 ± 0.6 ^a^	81.2 ± 0.9 ^a^	14.0 ± 1.6 ^a^	10.3 ± 0.7 ^a^
HPS	63.8 ± 0.5 ^c^	69.1 ± 1.0 ^d^	74.1 ± 1.0 ^d^	10.3 ± 0.9 ^c^	7.2 ± 1.7 ^c^
CAS	72.1 ± 0.6	76.2 ± 0.4	79.9 ± 0.6	7.8 ± 0.6	10.8 ± 0.8

Values under the same heading with identical superscript are not significantly different (*p* < 0.05).

**Table 4:** Density and Flow Properties of Native and Modified CMU-107 Starches *.

Sample	Density (g/cm^3^)		%CI	HR	AR
	Bulk	Tapped			
NS	0.56 ± 0.01 ^a^	0.73 ± 0.00 ^a^	23.49 ± 0.72 ^c^	1.31 ± 0.01 ^b^	24.60 ± 3.52 ^b^
PGS	0.43 ± 0.01 ^d^	0.59 ± 0.01 ^c^	28.50 ± 1.06 ^a^	1.40 ± 0.02 ^a^	27.39 ± 2.08 ^a^
CMS	0.51 ± 0.01 ^b^	0.65 ± 0.01 ^b^	21.09 ± 1.03 ^c^	1.27 ± 0.02 ^c^	18.33 ± 5.12 ^d^
CLCMS	0.46 ± 0.01 ^c^	0.58 ± 0.01 ^cd^	20.81 ± 1.27 ^d^	1.26 ± 0.02 ^c^	20.73 ± 2.15 ^c^
CLS	0.43 ± 0.00 ^d^	0.57 ± 0.00 ^d^	24.30 ± 0.30 ^b^	1.32 ± 0.01 ^b^	19.27 ± 2.66 ^cd^
HPS	0.44 ± 0.04 ^cd^	0.57 ± 0.02 ^cd^	23.50 ± 4.68 ^bc^	1.31 ± 0.08 ^bc^	22.08 ± 4.51 ^bc^
CAS	0.50 ± 0.04	0.67 ± 0.02	25.39 ± 3.27	1.34 ± 0.04	24.35 ± 2.18

* NS, native starch; PGS, pre-gelatinized starch; CMS, carboxymethyl starch; CLCMS, crosslinked carboxymethyl starch; CLS, crosslinked starch; HPS, hydroxypropyl starch; CAS, Cassava starch. Values under the same heading with identical superscript are not significantly different (*p* < 0.05).

## Data Availability

The data presented in this study are available on request from the corresponding author.
